# Building and Visualising Ordinations of Shape Data in R With *Morphospace*

**DOI:** 10.1002/ece3.71503

**Published:** 2025-06-19

**Authors:** Pablo S. Milla Carmona, Oscar E. R. Lehmann, William J. Deakin, Eduardo M. Soto, Ignacio M. Soto

**Affiliations:** ^1^ Bristol Palaeobiology Group, School of Earth Sciences University of Bristol Bristol UK; ^2^ Departamento de Ecología, Genética y Evolución, IEGEBA (CONICET−UBA), Facultad de Ciencias Exactas y Naturales Universidad de Buenos Aires Buenos Aires Argentina; ^3^ Sección Paleontología de Vertebrados (CONICET−MACN) Museo Argentino de Ciencias Naturales Buenos Aires Argentina

**Keywords:** geometric morphometrics, morphospaces, multivariate ordination methods, R package, shape variation, visualisation

## Abstract

Ordination is a critical step of geometric morphometrics that allows simplification of high‐dimensional shape spaces into low‐dimensional representations summarising shape variation. While this is routinely used to visualise the main patterns of morphometric data, the lack of a unified approach curbs researchers' ability to make the most of available ordination and visualisation methods. Here we introduce morphospace, an R package providing a streamlined approach to shape ordination and visualisation, intended to enhance the biological relevance of the analysis. This package integrates a series of tools into a workflow including (1) filtering variation associated with nuisance factors out from shape data; (2) building ordinations emphasising different types of biological signal and mapping shape variation into them; (3) projecting elements onto this morphospace, including empirical or theoretical shapes, shape clusters, morphometric axes, phylogenetic trees or performance landscapes; and (4) combining shape variation with non‐shape information to create alternative visualisations. We describe the basic mechanics and capabilities of morphospace, as well as its integration with other R packages, and briefly discuss the patterns it can help expose. Then, we showcase its applications by working three examples focused on phenomena commonly targeted in palaeontological and evolutionary research. The tools and workflow provided by morphospace facilitate the creation of publication‐ready visualisations to accompany results of dedicated statistical tests and enhance heuristic exploration of the patterns present in shape data and the underlying processes that generated them.

## Graphical abstract

Ordination and visualisation of shape data is a crucial step in the statistical analysis of morphology, but the lack of a unified approach prevents researchers from making the most of their data. We introduce morphospace, an R package aimed at filling this gap. The workflow, functionalities, and biological context of the morphospace tools are described and showcased through three worked examples.

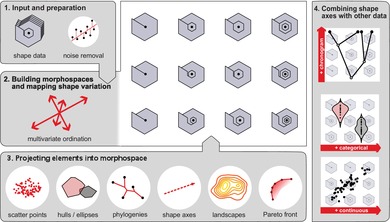

## 1 Introduction

Geometric morphometrics (GM) has become the standard framework for testing hypotheses about the processes that shape organismal form. The methods grouped under this umbrella—e.g., analysis of landmark (Adams et al. 2013) and semilandmark (Gunz and Mitteroecker 2013) data, or different flavours of Fourier analysis (e.g., elliptic Fourier analysis; Kuhl and Giardina 1982)—are underpinned by a fully operational definition of shape (i.e., the variation left after removing differences in position, orientation, and scale), the ability to analytically separate it from size, and the retention of the geometric information throughout the analyses (Rohlf and Marcus 1993; Mitteroecker and Gunz 2009). The modern shift toward open programming environments like R (R Core Team 2024) has fostered the development of specialized packages such as geomorph (Adams et al. 2021), Morpho (Schlager 2017), Momocs (Bonhomme et al. 2014), and shapes (Dryden 2023) which allow executing all the steps of GM analysis while significantly augmenting the variety and depth of GM operations available to the user.

While software for shape data acquisition and analytical processing has kept pace with advances in these areas, the lack of straightforward implementation for tools that summarize and visualize shape variation hinders their full integration into the standard GM pipeline. Principal Component Analysis (PCA) is routinely used to condense high‐dimensional shape spaces into Euclidean low‐dimensional approximations that are easier to interpret. However, recent advances in multivariate ordination now allow guiding this process using other continuous or categorical variables (Rohlf and Corti 2000; Mitteroecker and Bookstein 2011) or phylogenetic relationships (Revell 2009; Collyer and Adams 2021), thereby increasing the biological relevance of the analyses. At the same time, visualization options have evolved from traditional vectors, wireframes, and thin‐plate splines to include color maps, warped 2D templates, and 3D meshes that further facilitate interpretation of shape changes (Klingenberg 2013a; Mitteroecker and Schaefer 2022). Combined, these tools help distinguish the meaningful biological signal from background noise—useful when studying morphological processes that can be subtle or nuanced, enhancing the clarity with which underlying patterns emerge. Moreover, shape data ordination enables simulation and quantitative assessment of theoretical shapes beyond the empirical range, a distinct feature with important applications in evolutionary studies (e.g., Polly et al. 2016; Dickson et al. 2021; Deakin et al. 2022; Milla Carmona et al. 2022).

Yet, tools implementing these advances are either unavailable or scattered across the R landscape, hindering their integration. For example, geomorph and Morpho—the two main libraries for GM analysis in R—offer a variety of ordination analyses and graphical options but lack functionalities for mapping shape variation directly into the ordination space with enhanced graphical overlays. Momocs, the main GM library supporting outline analysis, allows representing 2D shape variation in different ways directly into the ordination axes (which we took inspiration from) but offers scant ordination alternatives. Built‐in capabilities for visualising elements beyond the standard scatter points, hulls, and Brownian Motion‐based phylogenetic relationships are limited, whereas R packages implementing sophisticated features—such as phylomorphospaces under complex evolutionary models (mvMORPH, Clavel et al. 2015) or landscapes reflecting functional trade‐offs (Morphoscape, Dickson et al. 2023)—are not explicitly designed to work with shape data, requiring additional customisation for integration into GM pipelines. Thus, in order to take full advantage of the arsenal of ordination methods and graphical tools available, pipelines must articulate a number of different analyses, methodologies, and formats—a difficult and time‐consuming task that can impact the quality of the study. Ultimately, we view these problems as a consequence of the lack of a unified approach towards the construction and representation of morphospaces.

**FIGURE 1:**
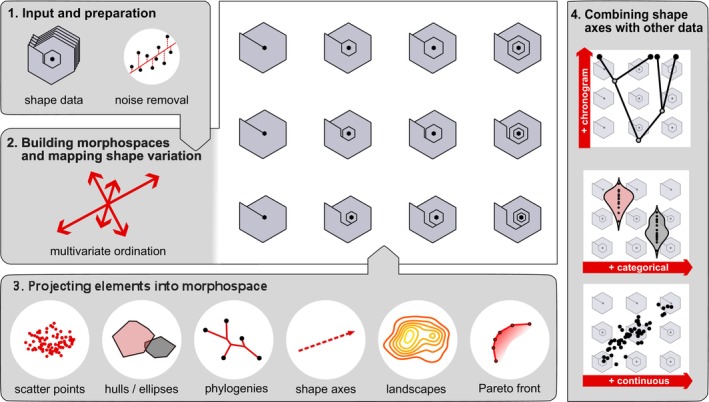
The morphospace workflow. (1) Basic input of shape data and optional refinement by removing variation associated to nuisance factors or covariates. (2) Selection of ordination method, calculation of background shape models, and projection into the ordination axes, i.e., the morphospace. (3) Projection of different elements into morphospace. (4) Combination of ordination axes with other types of data.

Here we introduce morphospace, a novel R package born from the necessity of streamlining the process of building and visualising multivariate ordinations of shape data, freely available at https://github.com/millacarmona/morphospace. morphospace builds upon the properties of shape ordination and the strengths of existing GM libraries to provide a structured and flexible approach to this task, allowing researchers to focus on the meaningful aspects of their analysis while tailoring visualisations to the specific biological question. This workflow (Figure 1) includes: (1) filtering shape data to remove noise from unwanted sources; (2) building and displaying ordinations using methods capable of emphasising different types of signal and representing morphology in varied ways; (3) projecting a range of different elements into the resulting ordination axes to highlight different kinds of patterns; and (4) combining the shape variation captured by individual ordination axes with non‐shape data. Below we describe this workflow in detail, walking through the functions involved, identifying points of integration with other R packages (functions from different packages are differentiated using the package::function notation), and discussing contexts in which these tools are most advantageous. Then, we showcase the capabilities of morphospace by working three case studies (included in the package as built‐in data sets), illustrating how these tools can be utilised to expose different morphometric patterns.

## 2 Package Overview

### 2.1 Input and Preparation

The basic input of the morphospace workflow is a set of shapes resulting from the normalization of raw landmarks and/or semilandmarks coordinates (2‐ or 3D anatomical loci, curves, surfaces) or elliptic Fourier coefficients (2D closed outlines) (Figure 1). These are the most common types of shape data used in the literature and can be readily obtained within the R environment using a number of functions (e.g., geomorph::gpagen, Morpho::procSym, or Momocs::efourier).

Methods for removing noise that might be masking the signal of interest prior to ordination are supported by morphospace::detrend_shapes. This function allows targeting variation associated with unwanted sources, which is then removed by estimating the shape residuals derived from a linear model (Klingenberg 1996, Revell 2009; supported options include stats::lm, geomorph::procD.lm, geomorph::procD.pgls, RRPP::lm.rrpp, mvMORPH::mvgls and mvMORPH::mvols), or alternatively, by projecting shapes into subspaces orthogonal to nuisance variables (Burnaby 1966). Other ways to refine shape variation include its decomposition into uniform and non‐uniform (e.g., using Morpho::relWarps; see Rohlf and Bookstein 2003) or symmetric and asymmetric components (e.g., using geomorph::bilat.symmetry; see Klingenberg et al. 2002). If instead variation associated with said source needs to be emphasized, theoretical shapes expected by the model (e.g., shapes representing groups' means or sitting on the regression line) can be obtained using morphospace::expected_shapes.

### 2.2 Building Morphospaces and Mapping Shape Variation

Once ready, shape variation can be summarised and depicted using morphospace::mspace, the function responsible for articulating ordination and visualisation methods (Figure 1). This function is compatible with ordination functionalities provided by geomorph, Morpho, Momocs, mvMORPH and phytools (as well as native ordination functions of morphospace; see below). The desired function can be specified using the FUN argument of morphospace::mspace (in which case shape data must be provided directly using the argument shapes); alternatively, an object containing the output of any of these functions can be fed to morphospace::mspace using its ord argument (an option that requires declaring the type of shape data being ordinated with the datype argument). Compatible ordination methods and functions implementing them are presented below according to the aspect of covariation that guides reorganisation of raw shape variation into new ordination axes.

#### 2.2.1 Internal Covariation

As noted previously, PCA (stats::prcomp, Momocs::PCA, geomorph::gm.prcomp) is conventionally employed to find the intrinsic directions of main shape variation of the sample, with PC1 capturing variation associated with the main processes affecting the sampled morphologies. This means that the orientation of PC1 will likely conflate multiple phenomena, especially in observational studies (e.g., allometry, temporal or geographical variation, interspecific divergence, etc.). Although this can be mitigated by removing shape variation associated with known nuisance factors (see above), the biological signal can be further emphasized using one of the several ordination methods that incorporate external information to capture specific components of shape variation.

#### 2.2.2 Covariation With External Variables

If the focus is placed on the morphological differences between groups of samples—e.g., taxa, sexes, ecomorphs, etc.—between‐groups PCA (bgPCA, see Mitteroecker and Bookstein 2011; morphospace::bg_prcomp, Morpho::groupsPCA) can be used to rotate the shape space such that the separation between groups defined a priori is maximised. Two‐blocks Partial Least Squares (PLS, see Rohlf and Corti 2000; morphospace::pls_shapes, Morpho::pls2B, geomorph::two.b.pls) is a similar method that can be used to capture the component of shape variation that covaries with an accompanying block of continuous variables—e.g., shape changes associated with variation in functional or environmental parameters, or even other aspects of morphology measured in the same individuals. If instead said component is to be minimised (e.g., to exclude pervasive sources of variation like allometry), its orthogonal subspace can be found using the Burnaby approach (morphospace::burnaby; Burnaby 1966).

#### 2.2.3 Phylogenetic Covariation

Comparative phylogenetic studies can use phylogenetic PCA to relegate the evolutionary homologous component of shape variation (pPCA, Revell 2009; phytools::phyl.pca, mvMORPH::mvgls.pca, geomorph::gm.prcomp). pPCA rotates the shape space to maximize the non‐phylogenetic component of shape variation, which can be useful to explore processes like adaptation, phenotypic plasticity, convergence, and other processes traditionally viewed as detached from phylogenetic history (although the full pPCA space preserves the original spatial relationships such that analyses of disparity or group separation still require phylogenetic correction; see Polly et al. 2013). If, on the other hand, the user wishes to emphasize the evolutionary homologous component, this can be achieved through phylogenetically aligned component analysis (PACA, see Collyer and Adams 2021; geomorph::gm.prcomp)—for example, to study instances of parallelism or macroevolutionary lines of least resistance.

For dimension reduction purposes (see Discussion), the number of relevant ordination axes can be determined using criteria like Kaiser's rule, or methods such as Horn's parallel analysis or Bookstein (2014)'s log‐likelihood ratio test‐based approach, which can be implemented in R using functions from different packages (e.g., nFactors::nScree, Raiche and Magis (2022), paran::paran, Dinno (2024), and Morpho::getMeaningfulPCs, respectively).

#### 2.2.4 Depicting Shape Changes

Following ordination, morphospace::mspace maps shape variation into ordination axes by computing and plotting a series of regularly‐spaced shape models (a popular strategy to depict shape variation, see MacLeod 2009). These can be further enhanced using ‘accessories’ like wireframes (lines interconnecting landmarks) and templates—a set of 2D or 3D curves or a 3D mesh to be warped via thin‐plate spline interpolation (Claude 2008)—, to aid visualisation of shape changes. The resulting shape models are deployed on the background of an empty plot made from a pair ordination axes—from here onwards, the ‘morphospace’.

### 2.3 Projecting Elements Into Morphospace

The ordination built with morphospace::mspace is used as a canvas in which different elements are projected in a layered fashion using the family of morphospace::proj_* functions (Figure 1) and the forward operator from magrittr (%>%; Bache and Wickham 2022). These serve to characterize the distribution of shape variation or highlight specific patterns.

#### 2.3.1 Shapes

**FIGURE 2:**
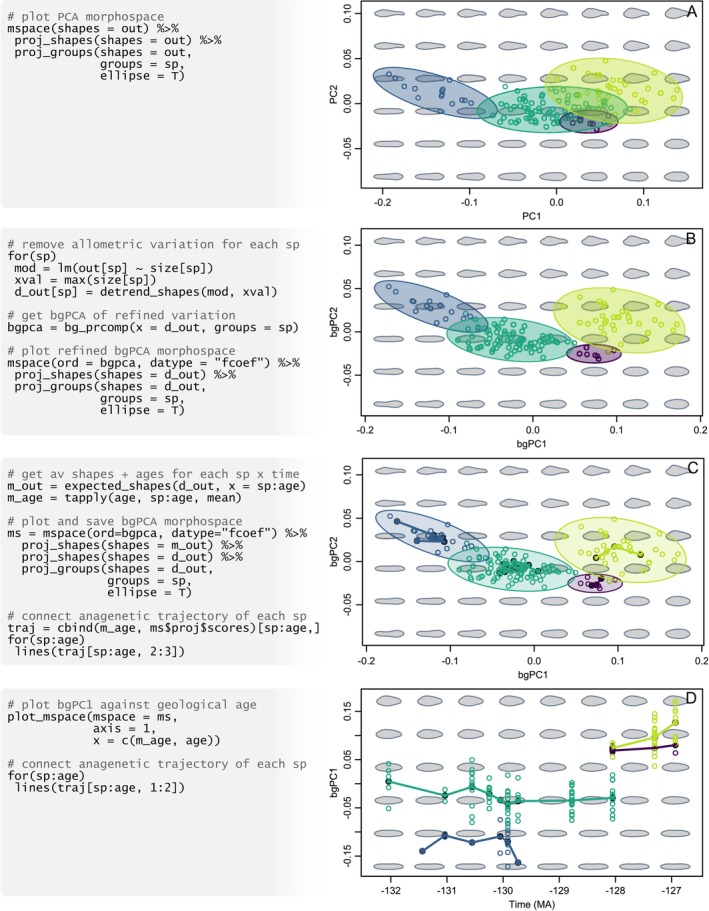
Exploring anagenetic evolution in *Ptychomya*. (A) Variation is condensed using PCA, and scatter points and 95% confidence ellipses representing *Ptychomya* species are projected. (B) Allometric variation is removed, prior maximising species differences using bgPCA. (C) Temporal trajectories are projected into bgPCA morphospace. (D) The first axis from bgPCA is combined with geological time to display anagenetic patterns of evolution. Pseudocode at the left is only illustrative; functioning code necessary to reproduce this figure is available at File S1.

**FIGURE 3:**
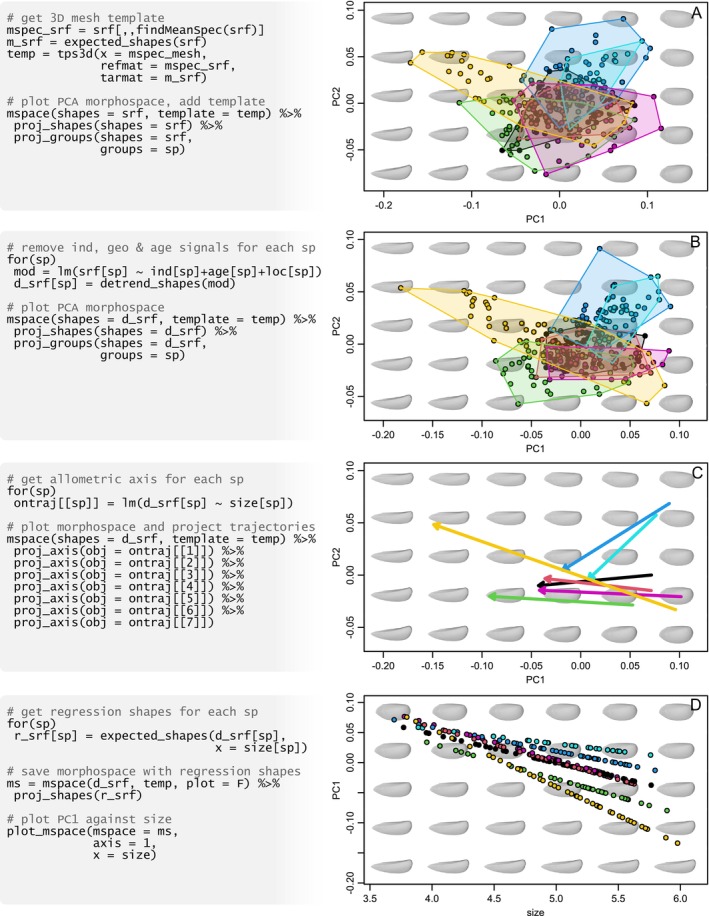
Exploring ontogenetic variation in *Steinmanella*. (A) A 3D mesh is warped into the template; variation is summarised using PCA, and scatter points and convex hulls representing the different species are projected. (B) Inter‐individual, geographic and stratigraphic variation are removed, prior to ordination of the filtered variation using PCA. (C) The (ontogenetic) allometric axis of each species is computed using linear models and projected into the refined morphospace. (D) The first axis from PCA is combined with log‐transformed size to further characterise species' trajectories. Pseudocode at the left is only illustrative; functioning code necessary to reproduce this figure is available at File S1.

The most basic of these functions is morphospace::proj_shapes, which projects one or more shapes as scatter points into an existing ordination. Most studies display points corresponding to the same sample of shapes used to build the ordination (e.g., Figures 2 and 3); however, ordination spaces allow projection of any compatible shape (i.e., measured using the same landmarks/semilandmarks or the same amount of Fourier harmonics), including partitions of the ordinated sample, empirical shapes not used during ordination, or theoretical shapes such as residual or mean shapes. This property can also be used to control the orientation of the ordination space—for example, resolution can be improved by excluding extreme shapes dominating the directions of maximum variation (e.g., a highly divergent clade) at the ordination stage and then projecting the full sample into the ‘unbiased’ axes.

#### 2.3.2 Groups

On the other hand, groups of shapes reflecting any biologically meaningful categorization of the data (e.g., clades, temporal levels, or experimental treatments) can be represented in morphospace using morphospace::proj_groups, which supports both convex hulls (appropriate for delimiting the full range of groups, but sensitive to outliers; e.g., Figure 3A,B) and confidence ellipses (useful to represent uncertainty associated to the groups' mean position or its covariance structure, but sensitive to sample size; e.g., Figure 2A–C).

#### 2.3.3 Shape Vectors

Other proj_* functions use theoretical shapes to depict more complex patterns or scenarios. For example, morphospace::proj_axis can be used to project a morphometric axis or vector (e.g., Figure 3C)—i.e., linear combinations of shape variables obtained through linear model fitting (stats::lm, geomorph::procD.pgls or mvMORPH::mvgls, among others) or multivariate ordination (any of the functions for multivariate ordination mentioned in the previous sections)—to represent patterns of shape change associated with phenomena like sexual dimorphism or evolutionary lines of least resistance.

#### 2.3.4 Phylogenies

**FIGURE 4:**
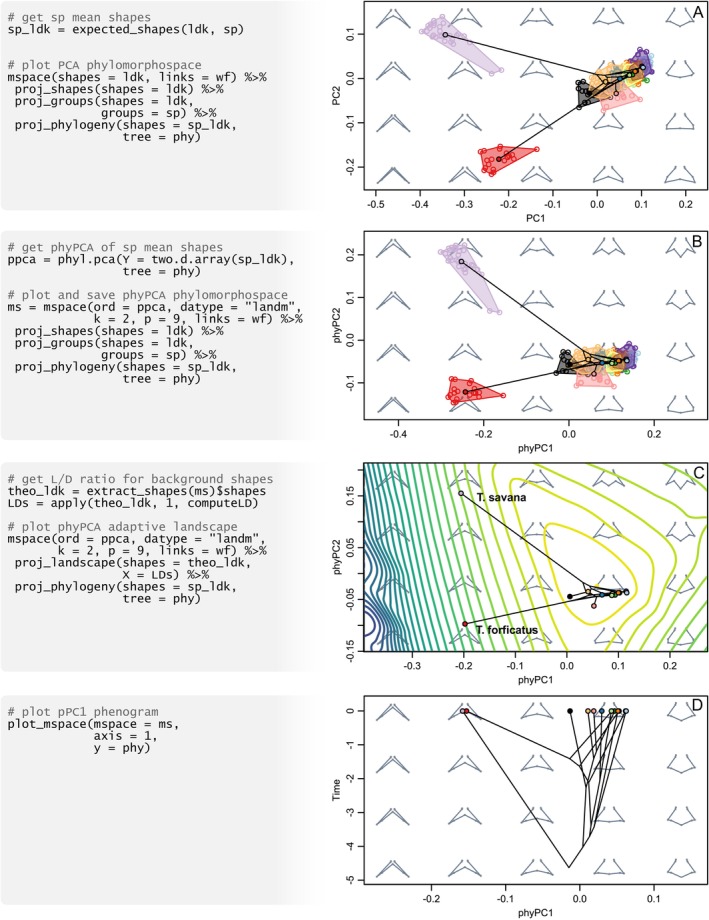
Exploring tail shape evolution in *Tyrannus*. (A) Species mean shapes are estimated; variation is summarised using PCA, and scatter points, convex hulls enclosing species, and phylogenetic relationships are projected. (B) Phylogenetically‐independent variation is emphasised using pPCA. (C) Functional values are calculated and then used to project an (empirical) landscape into pPC axes. (D) The first axis from pPCA is combined with a phylogenetic tree to create a phenogram. Pseudocode at the left is only illustrative; functioning code necessary to reproduce this figure is available at File S1.

Phylomorphospaces (Sidlauskas 2008) are a popular tool for representing shapes' phylogenetic relationships in comparative data sets, useful for studying evolutionary convergence, change in rates of morphological evolution, or differences in between‐clade morphospace occupation (Monteiro 2013). Phylogenetic relationships, including theoretical shapes estimated at the nodes under different models of phenotypic evolution (using functionalities from phytools and mvMORPH; Revell 2012; Clavel et al. 2015) can be projected into morphospace using morphospace::proj_phylogeny (e.g., Figure 4A–C).

#### 2.3.5 Landscapes

For functional studies, a surface relating shape and some measured component of fitness can be computed and projected using morphospace::proj_landscape (e.g., Figure 4C; landscapes computed with the Morphoscape package are also supported). Such landscapes provide a low‐dimensional approximation of the adaptive landscape (Arnold 2003; usually its performance‐related component) which, when extended beyond the range of empirical morphologies towards theoretical shapes charting unexplored regions of the morphospace, becomes a powerful heuristic tool for studying adaptive processes without making strong assumptions about realised performances. Both ‘empirical’ and ‘theoretical’ landscapes are supported, the latter requiring prior measurement of the functional properties of theoretical shapes mapping the surroundings of the realised morphospace (retrievable using morphospace::extract_shapes). If two or more functional metrics engaged in a trade‐off are being studied, these can be combined into a single Pareto optimality surface reflecting global performance (Deakin et al. 2022). The corresponding Pareto front, representing the subset of shapes that achieve the optimal balance between the studied functional metrics, can be projected with morphospace::project_pfront.

### 2.4 Combining Shape Axes With Other Data

Morphospaces resulting from the mspace %>% proj_* workflow can be regenerated, tweaked, and/or complemented with a legend or scale bar using morphospace::plot_mspace. In addition, this function allows mapping shape variation captured along individual ordination axes and combining them with two types of non‐shape information (Figure 1).

First, a phenogram showing the evolution of a clade through time and along individual ordination axes can be produced by providing a time‐calibrated phylogeny. Phenograms can help identify the extent of morphological convergence, the timing of evolutionary shifts, or patterns of extinction selectivity, among others. Second, ordination axes can be combined with accompanying variables to create ‘hybrid’ morphospaces expanding the catalogue of visual representations available to the user. These include bivariate plots showing covariation between shape and other continuous variables (e.g., size, time, functional metrics), as well as box‐ and violin‐plots for depicting the distribution of different levels of a categorical variable (e.g., species, clades, experimental groups) along individual axes of shape variation.

## 3 Worked Examples

### 3.1 Case Study 1: Anagenetic Evolution in Ptychomya

#### 3.1.1 Introduction and Data

*Ptychomya* is an extinct genus of veneroid bivalves with a rich fossil record in the Lower Cretaceous of Argentina, where four different species occur in abundance throughout a 5‐million year interval (132–127 MYA). This, coupled with the excellent geochronologic and biostratigraphic data available, offers an ideal opportunity to study patterns of anagenetic (i.e., within‐species) evolution. In order to estimate the relative frequency of different evolutionary modes (random walk, directional evolution and stasis) in these four species, Milla Carmona et al. (2018) measured variation in shell outline shape across a sample of 137 specimens—achieved through elliptic Fourier analysis and seven harmonics using Momocs::efourier. Apart from shape (out) and size (size), the data include taxonomic classification (sp) and the samples' geochronologic ages (age), estimated using a combination of available biostratigraphic and stratigraphic information and absolute dates (morphospace::shells).

#### 3.1.2 Data Cleaning

As the goal is to track temporal changes in average ‘static’ morphology, allometric variation is removed and the mean shape displaced to the shape predicted at maximum size, for each species separately, using morphospace::detrend_shapes. Average shapes for each combination of temporal level and species are obtained using morphospace::expected_shapes.

#### 3.1.3 Ordination

A PCA of raw (i.e., prior to noise removal) outline variation shows weak separation between *Ptychomya* species (Figure 2A). However, given the aim of this study, rotating the non‐allometric shape space to maximize interspecific differences using bgPCA (implemented using morphospace::bg_prcomp) represents a sensible choice (Figure 2C). Shell shapes are represented as silhouettes.

#### 3.1.4 Projections

Intra‐ and interspecific variation in shell outline is represented by projecting the full sample and 95% confidence ellipses using morphospace::proj_shapes and morphospace::proj_groups, respectively (Figure 2A–C). Temporal trajectories for each species are incorporated using previously calculated average shapes through morphospace::proj_shapes and graphics::lines.

#### 3.1.5 Combinations

Species' patterns of shape change through time are visualised by combining bgPC1 (accounting for more than 90% of the non‐allometric shape variation) and geological age into a hybrid morphospace with morphospace::plot_mspace (Figure 2D).

#### 3.1.6 Results and Discussion

These results indicate a strong nuisance effect of allometry; when this is removed and the interspecific differences enhanced, patterns of separation between species become much clearer. Projecting their temporal trajectories allows some idea of the anagenetic trajectory of each species, although reading how these unfold over time is still a difficult task—one that is facilitated by the shape‐time hybrid morphospace. The latter shows that most of these species experienced little or no net change over time (i.e., they undergo morphological stasis), with the exception of one species showing a short but noticeable trend towards shells with higher outlines. This interpretation is supported by the results of palaeontological time‐series analysis as well as the stratophenetic behavior of other morphological traits (Milla Carmona et al. 2018).

### 3.2 Case Study 2: Ontogenetic Variation in Steinmanella

#### 3.2.1 Introduction and Data

Trigoniid bivalves were a major group of marine invertebrates that went almost extinct during the end‐Cretaceous mass extinction, never to recover again. Milla Carmona et al. (2022) addressed the study of their ontogeny using the excellent fossil record of seven species of *Steinmanella* in the Lower Cretaceous of Argentina (approximately 140 MYA). In order to reconstruct and compare their ontogenetic trajectories, they collected longitudinal data from 67 specimens, measuring shape on each at different sizes for a total of 278 shapes (quantified using 3D surface sliding semilandmarks obtained with functionalities from Morpho). Apart from shape (srf) and size (size), the data set (morphospace::shells3D) also includes the individual each shape was measured from (ind), its taxonomic classification (sp), biostratigraphic age (age) and geographic provenance (loc), as well as the empirical 3D mesh of the specimen closest to the mean shape of the sample (mspec_mesh; found using geomorph::findMeanSpec).

#### 3.2.2 Data Cleaning

Since ontogenetic variation can be subtle and easily masked by other sources, inter‐specimen differences, geographic provenance, and geological age are removed for each species using morphospace::detrend_shapes. Shapes expected under a linear relationship with log‐transformed size were computed using morphospace::expected_shapes.

#### 3.2.3 Ordination

PCA of refined shapes is designed to maximize the ontogenetic allometric component of shell shape variation, as shown by its contrast with a PCA of raw data (Figure 3A,B). Shell shapes are represented using the 3D mesh of the sample's mean shape as a template, obtained by interpolating the transformation between the mean shape and its closest semilandmark configuration to warp the 3D mesh corresponding to the latter using Morpho::tps3d.

#### 3.2.4 Projections

The ontogenetic allometric variation of each species is projected and delimited with convex hulls using morphospace::proj_shapes and morphospace::proj_groups (Figure 3A,B). Subsequently, ontogenetic axes—estimated by fitting a linear model of refined shape variation on log‐transformed size with stats::lm—are projected into morphospace using morphospace::proj_axes (Figure 3C).

#### 3.2.5 Combinations

PC1 (52% of the refined shape variation) is combined with log‐transformed size to create a hybrid morphospace in which theoretical shapes sitting on each species' regression lines are projected using morphospace::plot_mspace (Figure 3D).

#### 3.2.6 Results and Discussion

While data collection and analysis maximise ontogenetic variation, estimating and visualising shape‐size regressions in morphospace greatly aid interpretation of ontogenetic changes. This hints at the existence of two distinct groups of ontogenies within *Steinmanella*—the two species in the upper‐right region of the morphospace reaching shorter rectangular shells, and the rest of the species whose shells become longer and triangle shaped. Visualisation of PC1 against log‐transformed size shows that, while the final shape of the first group resembles an immature shape of the second, they attain it at mostly the same final size—with the exception of the two larger species, one of which also displays the longer shape trajectory. These conclusions are supported by results of segmented regression and trajectory analysis (Milla Carmona et al. 2022).

### 3.3 Case Study 3: Convergent Tail Evolution in Tyrannus

#### 3.3.1 Introduction and Data

*Tyrannus* is a passerine genus of 13 small species native to the Americas. Most of these are kingbirds showing unremarkable rounded or notched tails; however, they also include two convergent species of flycatchers ( *T. savana* and *T. forficatus* ) with exaggerated deep‐forked tails, which are considered the result of sexual selection. Since bird tail morphology reflects the balance between the latter and natural selection on aerodynamic performance, flycatcher tails could be expected to deviate from the functional optimum, performing poorly as flight devices. We explore this prediction under a performance landscape approach using data from Fasanelli et al. (2022; morphospace::tails)—281 tail shapes from all *Tyrannus* species measured using nine 2D landmarks (ldk; previously superimposed using geomorph::gpagen), a wireframe connecting them (wf), their classification (sp), and a phylogenetic tree taken from the literature (phy; see references therein)—and accounting for aerodynamic performance using the lift‐to‐drag ratio (LD; Thomas 1993).

#### 3.3.2 Data Cleaning

Tail feathers are not fixed relative to each other, introducing noise in the form of asymmetric variation. Therefore, the symmetric component of variation is first isolated using geomorph::bilat.symmetry and retained for further analysis. Mean shapes for each species were obtained using morphospace::expected_shapes.

#### 3.3.3 Ordination

A first ordination attempt using PCA resulted in a morphospace dominated by the marked difference between flycatchers and kingbirds, running the risk of masking more subtle patterns of variation (Figure 4A). A second ordination is thus performed using pPCA through phytools::phyl.pca, to prioritize the non‐homologous component of kingbird tail variation (Figure 4B)—an appropriate choice when dealing with non‐historical aspects of adaptation such as biomechanical performance. Tail shapes are represented using wireframes.

#### 3.3.4 Projections

Phylogenetic relationships between *Tyrannus* species are projected into morphospace using morphospace::proj_phylogeny. Species' intraspecific variation and their ranges are represented by projecting the full sample and convex hulls with morphospace::proj_shapes and morphospace::proj_groups, respectively (Figure 4A,B). Aerodynamic performance is mapped into morphospace by projecting a landscape—computed from LD scores measured in background shapes—using morphospace::proj_landscape (Figure 4C).

#### 3.3.5 Combinations

pPC1 is combined with the time‐calibrated phylogeny to create a phenogram (Figure 4D) using morphospace::plot_mspace.

#### 3.3.6 Results and Discussion

The morphospace maximising non‐phylogenetic variation is distinctly oriented, although divergence between kingbirds and flycatchers still dominates the first two axes. The phenogram shows that convergence is maximised along the main non‐phylogenetic component, consistent with a non‐historical functional driver. When projecting the ‘theoretical’ performance landscape, it becomes evident that kingbirds are sitting on an aerodynamic optimum, whereas flycatchers diverge from it advancing into regions of the morphospace associated with poor performances—a pattern consistent with sexual selection as evolutionary driver. However, these two species are not complete functional equivalents: whereas *T. forficatus* descends the steeper slope of the landscape, *T. savana* follows a gentler path that retains some aerodynamic performance, suggesting relatively stronger aerodynamic pressures.

## 4 Discussion

Morphospaces are mathematical spaces resulting from the combination of variables measuring organismal morphology, the number and nature of which determine the dimensionality and geometric properties of the derived space (Mitteroecker and Huttegger 2009). Ordination allows simplifying the high‐dimensional morphometric patterns embedded in shape data into a few synthetic axes, having two main applications in the context of GM analysis.

First, a number of lower axes can be discarded to meet the requirements of statistical methods—for example, by keeping only the first axis to feed univariate methods, or by discarding the last axes to compensate for rank deficiencies introduced by procedures like shape normalisation or noise removal, which preclude direct analysis of shape variables using standard parametric multivariate tests (Zelditch et al. 2012). If a more nuanced approach to dimension reduction is required (e.g., to deal with the ‘curse of dimensionality’; Mitteroecker and Schaefer 2022), this can be achieved by applying any of the well established criteria for determining the true underlying dimensionality of multivariate data sets (see above). In some instances, ordination can impose some degree of information loss; for example, the number of resulting axes can get truncated to the number of observations when these are fewer than the number of original variables, to the minimum number of variables in either block for PLS, or to the number of groups—1 for bgPCA. The extent of this loss will depend on the amount of total shape variation accounted for by the external variables.

The second and most common use of ordination in GM is producing graphical representations supporting the results from more rigorous statistical tests. In this sense, we believe morphospace is a useful resource that equips the user with means to design insightful, publication‐ready visualisations of shape data patterns. However, this use of morphospaces also highlights their value as heuristic tools for exploring or illustrating the evolutionary and ecological processes shaping phenotypic diversity (Mitteroecker and Huttegger 2009; Serrelli 2015; Polly and Motz 2016). In GM, every possible location in a morphospace represents a distinct shape, even if not present in the empirical data set. Therefore, mechanisms generating or sorting shape variation can be represented in condensed morphospaces as two‐ or three‐dimensional objects linking a number of empirical and/or theoretical morphologies, thus giving an entity of sorts to abstract processes that can be difficult to grasp otherwise. Notable applications of this rationale include the trajectory analysis framework developed by Adams and Collyer (2009), the phylomorphospace approach of Sidlauskas (2008) or the implementation of performance landscapes described in Polly et al. (2016). We believe the tools and workflow of morphospace are valuable in this sense too, providing a platform that researchers can use to explore their data and sharpen their scientific hypotheses.

On the other hand, obtaining meaningful insight from morphospaces also requires awareness of their limitations. Dimension reduction procedures can introduce potential distortions necessitating correction and/or consideration—including the spurious agglomeration of observations in the center of bivariate PCA morphospaces (Polly 2023) or the artificial separation between groups or covariation between blocks arising in bgPCA and PLS when there are more cases than variables (which can be alleviated using leave‐one‐out cross validation; Rohlf 2021). Also, not all the geometric properties of morphospaces are equally robust to departures from Euclidean behavior caused by some attributes of GM methods (Mitteroecker and Huttegger 2009; Mitteroecker and Schaefer 2022), which can complicate the comparison of distances or angles calculated over different dimensions. Still, a number of properties can be reliably interpreted, including patterns of group overlap and separation, parallelism (or lack of thereof) of trajectories, or ratios of volumes (see Mitteroecker and Schaefer 2022). While these issues warrant careful interpretation of morphospaces (especially if retaining only the first two or three axes), they do not negate their heuristic value as long as the statistical consistency of morphometric patterns is assessed using adequate dimensionality and dedicated statistical tests (e.g., multivariate linear models, Zelditch et al. 2012; trajectory analysis, Adams and Collyer 2009; integration and modularity analysis, Klingenberg 2013b; disparity analysis, Guillerme et al. 2020; morphological convergence analysis, Castiglione et al. 2019; evolutionary model fitting, Hunt 2006; Clavel et al. 2015; analysis of asymmetry, Savriama and Klingenberg 2011; among many others), which can be readily implemented using a number of different R packages.

## 5 Conclusion

Despite the development of ordination and visualisation methods enabling the construction of morphospaces tailored to specific research questions, the lack of a unified implementation has so far hindered their widespread utilisation beyond conventional alternatives. morphospace addresses this gap by providing a workflow devised to (1) integrate these diverse methods and data into a streamlined pipeline, (2) make the user mindful of the methodological choices involved, (3) ensure compatibility with R libraries widely used in GM analysis, and (4) produce intuitive, compelling visualisations ready for publication. This package, together with the methodological overview provided in this paper, is ultimately intended to help GM practitioners make the most of their data by enhancing both the discovery and communication of (geometric) morphometric patterns.

## Author Contributions

**Pablo S. Milla Carmona:** conceptualization (equal), data curation (equal), formal analysis (equal), funding acquisition (equal), methodology (equal), resources (supporting), software (lead), supervision (equal), validation (lead), visualization (lead), writing – original draft (lead), writing – review and editing (equal). **Oscar E. R. Lehmann:** data curation (equal), formal analysis (equal), methodology (equal), software (equal), validation (equal), writing – review and editing (equal). **William J. Deakin:** formal analysis (equal), methodology (equal), software (equal), validation (equal), writing – review and editing (equal). **Eduardo M. Soto:** conceptualization (equal), data curation (equal), formal analysis (equal), funding acquisition (equal), project administration (equal), validation (equal), writing – review and editing (equal). **Ignacio M. Soto:** conceptualization (equal), data curation (equal), funding acquisition (equal), methodology (equal), project administration (equal), supervision (equal), writing – original draft (equal), writing – review and editing (equal).

## Conflicts of Interest

The authors declare no conflicts of interest.

## Supporting information


File S1.

## Data Availability

All the data used are available at https://github.com/millacarmona/morphospace/tree/main/data.
